# Effect of Thermal Vacancy on Thermodynamic Behaviors in BCC W Close to Melting Point: A Thermodynamic Study

**DOI:** 10.3390/ma11091648

**Published:** 2018-09-07

**Authors:** Ying Tang, Lijun Zhang

**Affiliations:** 1 School of Materials Science and Engineering, Hebei University of Technology, Tianjin 300130, China; 2 State Key Laboratory of Powder Metallurgy, Central South University, Changsha 410083, China

**Keywords:** thermal vacancy, thermodynamics, heat capacity, bcc tungsten

## Abstract

As temperature increases, the thermal vacancy concentration in pure metals dramatically increases and causes some strongly non-linear thermodynamic behaviors in pure metals when close to their melting points. In this paper, we chose body-centered cubic (bcc) W as the target and presented a thermodynamic model to account for its Gibbs energy of pure bcc W from 0 K to melting point by including the contribution of thermal vacancy. A new formula for interaction part was proposed for describing the quadratic temperature behavior of vacancy formation energy. Based on the experimental/first-principles computed thermodynamic properties, all the parameters in the Gibbs energy function were assessed by following the proposed two-step optimization strategy. The thermodynamic behaviors, i.e., the strong nonlinear increase for temperature dependence of heat capacities at high temperatures and a nonlinear Arrhenius plot of vacancy concentration, in bcc W can be well reproduced by the obtained Gibbs energy. The successful description of thermal vacancy on such strongly non-linear thermodynamic behaviors in bcc W indicates that the presently proposed thermodynamic model and optimization strategy should be universal ones and are applicable to all other metals.

## 1. Introduction

**Figure 1:**
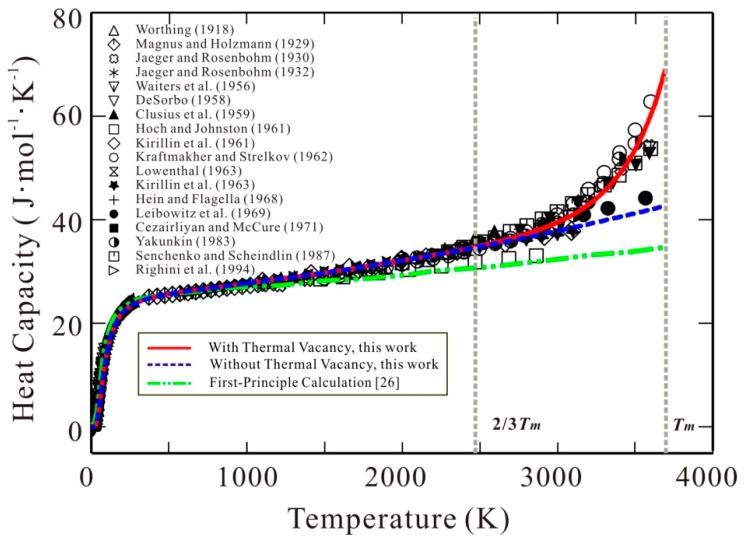
Heat capacity of body-centered cubic (bcc) W as a function of temperature. Symbols: Experimental data [7,8,9,10,11,12,13,14,15,16,17,18,19,20,21,22,23]. Solid line (red): Calculated results according to the presently established Gibbs energy for bcc W with thermal vacancy contribution. Dashed line (blue): Calculated results according to the presently established Gibbs energy for defect-free bcc W without thermal vacancy contribution. Dotted line (green): First-principles calculation [19], which does not include thermal vacancy contribution.

Thermal vacancy is the simplest but extremely important structural defect in pure metals. As temperature increases, the thermal vacancy concentration in pure metals dramatically increases, and makes an apparent contribution to different physical quantities of materials, such as heat capacity, melting point, diffusivity, thermal conductivity, and so on [1,2,3]. Taking body cubic centered (bcc) W that has been proposed for use in the divertor of future fusion devices [4,5], for example, its thermal vacancy concentration can be larger than 0.02 at its melting point [6,7]. With such a large thermal vacancy concentration, the heat capacity of bcc W over the high-temperature region shows a strong non-linear increase, as demonstrated by most of the experimental data available in the literature [7,8,9,10,11,12,13,14,15,16,17,18,19,20,21,22,23] and plotted in Figure 1. Such strongly non-linear behavior on heat capacity in the region close to the melting point is related to the dramatic increase of thermal vacancy. The recent first-principles computed heat capacities of bcc W [24] are also superimposed in Figure 1 for a comparison with the experimental data. As clearly seen in Figure 1, the first-principles calculations only taking the harmonic/anharmonic vibration and electronic excitation into account [24] cannot accurately predict the heat capacity of W with such a non-linear increase over the high-temperature region. This fact indicates that the thermal vacancy contribution to heat capacity is noticeable at high temperatures. In addition to heat capacity, the thermal vacancy also shows obvious influence on self-diffusivity of bcc W. The Arrhenius plot for measured self-diffusivities in bcc W over a wide temperature range shows significant curvature [25]. Kraftmakher [3] pointed out that one probable reason for such curvature in self-diffusivities of bcc W lies in the fact that the concentration of thermal vacancy has a similar temperature dependence.

Furthermore, recent theoretical predictions [26,27] show that formation entropy of vacancies is not constant as commonly assumed but increases with temperature, resulting in highly nonlinear temperature dependence in the formation energy. This point may naturally explain the strongly non-linear increase of the heat capacity at high temperatures and curvature in Arrhenius plot of vacancy concentration. Thus, in order to quantitatively describe the effect of thermal vacancy on these strongly non-linear thermodynamic behaviors in bcc W, accurate prediction of the temperature-dependent thermal vacancy formation energy is the prerequisite, as is the major task of this paper.

## 2. Thermodynamic Model for bcc W with Thermal Vacancy

By considering the contribution of both thermal vacancy and W atoms, the molar Gibbs free energy of pure element W in consistency with Compound Energy Formalism (CEF) can be described as [28,29]:

(1) $$G_{m}=\frac{1}{y_{W}}[y_{W}G_{W}+y_{\mathrm{va}}G_{\mathrm{va}}+RT(y_{W}\ln y_{W}+y_{\mathrm{va}}\ln y_{\mathrm{va}})+y_{W}y_{\mathrm{va}}\Omega ]\;$$

where *R* is the gas constant, *T* the absolute temperature, while *y*_w_ and *y*_va_ the site fractions of species W and thermal vacancy, respectively. The summation of *y*_w_ and *y*_va_ should be unity. *G*_W_ is the molar Gibbs energy of the defect-free element W, *G*_va_ the molar Gibbs energy of a virtual empty bcc lattice, while *Ω* is the interaction parameter. 

### 2.1. Expression for G_W_

The molar Gibbs energy of the defect-free element W, i.e., *G*_W_, from 0 K to melting point in Equation (1) can be expressed by using the following physical model [30]:

(2) $$G_{W}=E_{0}+\frac{3}{2}R\theta_{E}+3RT\ln[1-\exp(-\frac{\theta_{E}}{T})]-\frac{a}{2}T^{2}-\frac{b}{6}T^{3}-\int_{0}^{T}[\int_{0}^{T}\frac{C_{p}^{\mathrm{mag}}}{T}dT]dT\;$$

where *E*_0_ is the total energy of ferromagnetic pure W at 0 K which can be directly obtained from the first principles calculations, while the second term is the energy of zero-point lattice vibration [31,32] with *θ*_E_ as the Einstein temperature. The remaining four terms are derived from the integration of the heat capacity model for pure metals proposed in 1995′s Ringberg Workshop [30]:

(3) $$C_{p}{}^{\mathrm{Pure}\;W}=3R{\left(\frac{\theta_{E}}{T}\right)}^{2}\frac{e^{\theta_{E}/T}}{{\left(e^{\theta_{E}/T}-1\right)}^{2}}+aT+bT^{2}+C_{p}^{\mathrm{mag}}\;$$

The first term in Equation (3) is the Einstein heat capacity, which is mostly contributed from harmonic vibration. The second term *aT* in Equation (3) is related to electronic excitations and low-order anharmonic corrections, while the third term *bT*^2^ contains the high-order anharmonic corrections. $C_{p}^{\mathrm{mag}}$ is the contribution from the magnetic ordering. It should be noted that, in order to obtain the heat capacities for defect-free W, the coefficients *a* and *b* need to be evaluated by considering the experimental data without any contribution from thermal vacancy.

### 2.2. Expression for G_va_

For the molar Gibbs energy of a virtual empty bcc lattice, *G*_va_, in Equation (1), it is very difficult to define its standard reference value in a physical, meaningful way [33]. In order to make the balance between the different terms in Equation (1) over the wide temperature range, *G*_va_ is usually suggested to be proportional to temperature [27]. Besides, it would be desirable to set a general value for *G*_va_, which is independent of the elements, if for a universal usage for i.e., developing a common thermodynamic database. In 2014, Franke [28] pointed out that the value of *G*_va_ should be larger than a critical value (i.e., (ln2−1/2)RT) to ensure a unique equilibrium state. In other words, the critical value can also ensure the second derivative of Gibbs energy $\partial^{2}G_{m}/\partial y_{\mathrm{va}}{}^{2}$ to be positive at any temperatures, resulting in a unique equilibrium state. Moreover, such a critical value was obtained from a thorough mathematical analysis by Franke [28], and should thus serve as a common one which does not depend on the types of elements or phases.

In this work, the molar Gibbs energy of vacancy is simply set to 0.2*RT*, which is slightly larger than the critical value, as also proposed in Reference [28]. 

### 2.3. Expression for Interaction Parameter Ω

As for the interaction parameter Ω, one needs to figure out its relation with the non-linear concentration of thermal vacancy before proposing its expression. Based on Equation (1), for the equilibrium state of bcc W, one can have,

(4) $$\frac{\partial G_{m}}{\partial y_{\mathrm{va}}}=\frac{1}{{\left(1-y_{\mathrm{va}}\right)}^{2}}G_{\mathrm{va}}+\frac{1}{{\left(1-y_{\mathrm{va}}\right)}^{2}}RT\ln y_{\mathrm{va}}+\Omega =0\;$$

by applying the constraint *y*_w_ + *y*_va_ = 1. From Equation (4), the equilibrium concentration of thermal vacancy satisfies the following relation:

(5) $$y_{\mathrm{va}}=\exp(-\frac{G_{\mathrm{va}}+\Omega {\left(1-y_{\mathrm{va}}\right)}^{2}}{RT})\;$$

Meanwhile, the vacancy concentration can be also expressed as

(6) $$y_{\mathrm{va}}=\exp(-G_{\mathrm{va}}^{f}/RT)=\exp(-(H_{f}-TS_{f})/RT)\;$$

where $G_{\mathrm{va}}^{f}$ is the vacancy formation energy, *H*_f_ and *S*_f_ are the formation enthalpy and entropy of thermal vacancies, respectively. Comparing Equation (5) with Equation (6), it is easy to see that the value *G*_va_ *+ Ω* approaches to the formation energy of thermal vacancy in the limit of negligible vacancy concentrations. However, it seems to be very difficult and even impossible to separate the contributions of *G*_va_ and Ω from the formation energy of thermal vacancy [33]. The assumption of a constant entropy of formation of the thermal vacancy would introduce linear temperature dependence in $G_{\mathrm{va}}^{f}$ and hence lead to a constant prefactor to the Arrhenius plot of thermal vacancy concentration. However, for the Arrhenius plot of vacancy concentration with curvature behavior, i.e., *y*_va_ in bcc W, such assumption cannot give a reasonable description especially in the high temperature range. In order for an accurate thermodynamic description, the nonlinear temperature behavior of the vacancy formation energy needs to be considered. In a first approximation, we here assume a linear temperature dependence for *S*_f_; therefore, a temperature quadratic term will be included in *H*_f_ as well as in $G_{\mathrm{va}}^{f}$ due to the thermodynamic relation $(\partial H_{f}/\partial T{)}_{P}=T(\partial S_{f}/\partial T{)}_{P}$. Moreover, we simply use the linear temperature dependence of $G_{\mathrm{va}}^{}$ in the present work, i.e., 0.2*RT*, following the work of Franke [28]. In order to describe the quadratic temperature behavior of vacancy formation energy, we thus propose an expression for interaction parameter *Ω* in Equation (1):

(7) $$\Omega =A+BT+CT^{2}\;$$

Here, *A*, *B* and *C* are the parameters, which can be assessed on the basis of the experimental data, like thermal vacancy concentration, and/or heat capacities at high temperatures. Substituting Equation (7) into Equation (5), one can easily see that the proposed interaction expression is able to describe the curvature between $\log y_{\mathrm{va}}$ and 1/*T*.

Moreover, based on the molar Gibbs energy expression for bcc W together with Equations (4) and (5), its heat capacity can be derived as

(8) $$C_{p}=C_{p}{}^{\mathrm{Pure}\;W}-y_{\mathrm{va}}T\frac{\partial^{2}\Omega }{\partial T^{2}}+(\Omega -T\frac{\partial \Omega }{\partial T})\frac{\partial y_{\mathrm{va}}}{\partial T}\;$$

in which $C_{p}{}^{\mathrm{Pure}\;W}$ represents the heat capacity of defect-free element W. The second and third terms on the right-hand side of Equation (8) denote the effects of thermal vacancy on heat capacity. When submitting Equation (7) into Equation (8), it can be found that the effects of thermal vacancy on heat capacity will become obvious with the increase of the temperature, which is excepted to describe the strongly non-linear behavior of thermodynamic properties near the melting point.

## 3. Results and Discussion

The heat capacity of bcc W was experimentally measured by several groups [7,8,9,10,11,12,13,14,15,16,17,18,19,20,21,22,23] from 0 K to melting point, as shown in Figure 1. The heat capacity shows a rapid increase at high temperatures especially close to the melting point. Moreover, the enthalpy increment of bcc W was also measured over a wide temperature range [14,34,35,36,37,38,39]. Additionally, the concentration of thermal vacancy in bcc W was also experimentally investigated at melting temperature [6,7]. Although no experimental vacancy concentration as a function of temperature was reported in the literature, Kraftmakher [3] derived the equilibrium vacancy concentrations in bcc W from the heat capacities with nonlinear increase [7]. 

The quantification of all the parameters in the molar Gibbs energy of bcc W (Equation (1)) can be divided into two steps. The first step is to fix the molar Gibbs energy of the defect-free element W, i.e., *G*_W_. Theoretically, the contribution of thermal vacancy should be excluded for the heat capacity and Gibbs energy of defect-free bcc W. However, it is also very difficult to separate the contribution of thermal vacancy for the experimental heat capacities completely in reality. The accurate first-principles calculations might provide the data for defect-free bcc W, but the current first-principles computed heat capacities of defect-free bcc W available in the literature [24] are lower than the experimental data above 1500 K (see Figure 1). As pointed out by Kraftmakher [3], the vacancy contribution to specific heat becomes visible only at temperatures above about two-thirds of the melting temperature (i.e., 2463 K for bcc W). Moreover, for bcc W, the magnetic effects on heat capacity can be ignored. In addition, Walford [40] experimentally measured the Debye temperature (*θ*_D_) of bcc W, which was reported to be 377 K. The corresponding Einstein temperature (*θ*_E_) can be evaluated as 269.2 K based on the relation $\theta_{E}\approx 0.714\theta_{D}$ [41]. Then, the heat capacity of defect-free bcc W was obtained by fitting the experimental heat capacities and enthalpy increments below 2/3 *T*_m_. Because there is no measured total energy (*E*_0_ in Equation (8)) of bcc W at 0 K in the literature, the first-principles calculated result due to Wang et al. [42] was directly used here to get the expression for Gibbs energy of defect-free bcc W. The second step is to assess the three coefficients constituting the interaction parameter, Ω, based on the experimental heat capacities over the range of 2/3*T*_m_~*T*_m_. The finally obtained thermodynamic parameters in Gibbs energy expression for bcc W are listed in Table 1.

Figure 1 shows the calculated heat capacity for bcc W using the presently obtained Gibbs energy expression in comparison with the experimental data [7,8,9,10,11,12,13,14,15,16,17,18,19,20,21,22,23]. As can be seen in the figure, excellent agreement between the calculations and the experiments is obtained. For a comparison, the calculated heat capacity of defect-free bcc W is also superimposed in Figure 1. The deviation between the heat capacity with and without thermal vacancy is quite obvious at high temperatures, and can reach 26.5 J (mol K)^−1^ at melting temperature. It indicates that the thermal vacancy has a significant effect on the thermodynamic properties of the pure metals. Moreover, one can clearly see the strongly non-linear behavior of heat capacities of bcc W, i.e., the dramatic increase of heat capacity at high temperatures close to melting point can be well reproduced by the presently obtained Gibbs energy of bcc W with thermal vacancy.

**Figure 2:**
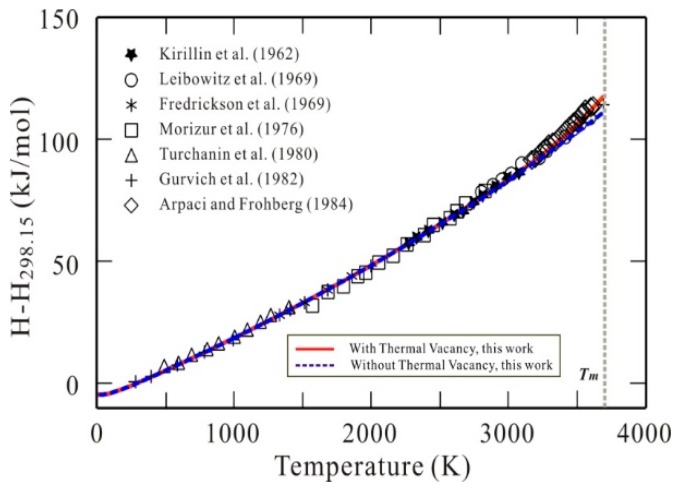
Heat contents (*H* − *H*_298.15_) of bcc W as a function of temperature. Symbols: Experimental data [14,34,35,36,37,38,39]. Solid line (red): Calculated results according to the presently established Gibbs energy for bcc W with thermal vacancy contribution. Dashed line (blue): Calculated results according to the presently established Gibbs energy for defect-free bcc W without thermal vacancy contribution.

Figure 2 displays the presently calculated heat contents (*H* − *H*_298_) of bcc W with and without thermal vacancy along with experimental data [14,34,35,36,37,38,39]. As shown in Figure 2, the calculated results with thermal vacancy reproduce the reported data very well, while the results without thermal vacancy show a small deviation from the experimental data at the high temperature range. It indicates that the present thermodynamic description can well describe the thermal vacancy contribution to the heat contents, though the thermal vacancy contribution to heat content at high temperatures is relatively small compared with that to heat capacity. 

**Figure 3:**
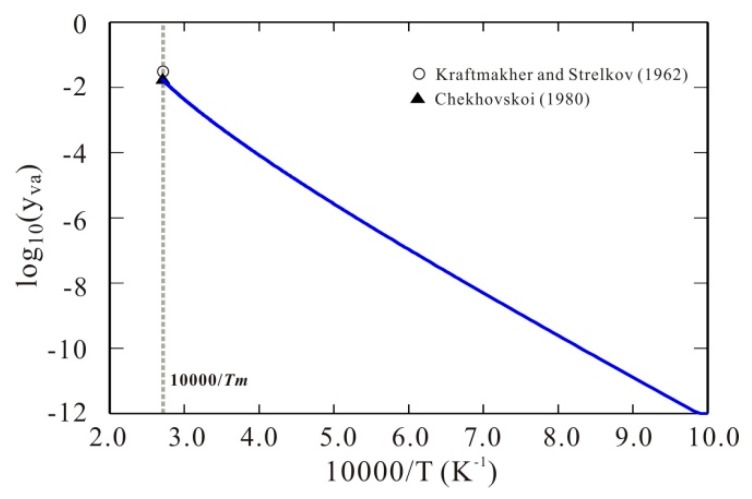
Arrhenius plots of thermal vacancy concentration of bcc W. Symbols: Experimental data at melting point from [9,10]. Solid line: Calculated thermal vacancy concentration of bcc W according to the presently established Gibbs energy for bcc W. A clear curvature is obtained for the Arrhenius plot of thermal vacancy concentration of bcc W.

Figure 3 presents the model-predicted 10-base logarithm values of thermal vacancy concentration of bcc W as a function of 10,000/T due to the obtained Gibbs energy of bcc W with thermal vacancy, compared with the experimental data at melting temperature [6,7]. As can be seen in the figure, the model-predicted thermal vacancy concentration of bcc W is 0.018, which agrees well with the experimental data [6,7]. Moreover, as expected, such Arrhenius plot of vacancy concentration shows a clear curvature, which well reproduces the non-linear behavior stated by Kraftmakher [3] and also theoretically predicted by Koning et al. [26] and Glensk et al. [27].

## 4. Conclusions

In this paper, a thorough thermodynamic study on the effect of thermal vacancy on strongly non-linear thermodynamic behaviors in bcc W close to its melting point was performed. By considering the contribution of thermal vacancy, a thermodynamic model was presented for describing the Gibbs energy of pure bcc W from 0 K to melting point. A new formula for interaction part as well as a pragmatic two-step optimization strategy was proposed. With the obtained Gibbs energy of pure bcc W assessed from the experimental/first-principles computed thermodynamic quantities, the thermodynamic behaviors in bcc W, including the strongly nonlinear temperature-dependence heat capacity close to the melting point, and a nonlinear Arrhenius plot of vacancy concentration were well reproduced. It is anticipated that the presently proposed thermodynamic model and optimization strategy for bcc W should be universal ones and are applicable to all other metals.

## Figures and Tables

**Table 1:** List of the evaluated thermodynamic parameters for bcc W.

Parameters	Values (Gibbs Energy in J/mol-atom; T in Kelvin)
$G_{W}$	$\begin{array}{l} E_{0}+\frac{3}{2}R\theta_{E}+3RT\ln(1-\exp(-\frac{\theta_{E}}{T}))-1.085\times 10^{-3}T^{2}-1.1835\times 10^{-7}T^{3}\\ \left(E_{0}=-1228665.43,\begin{array}{cc} & \theta_{E}=269.2 \end{array}\right) \end{array}$
$G_{\mathrm{Va}}$	+0.2RT
Ω	$+229615.89+12.73T-1.1274\times 10^{-2}T^{2}$
